# High level of sperm competition may increase transfer of accessory gland products carried by the love dart of land snails

**DOI:** 10.1002/ece3.3385

**Published:** 2017-11-17

**Authors:** Monica Lodi, Alexandra Staikou, Ruben Janssen, Joris M. Koene

**Affiliations:** ^1^ Section of Animal Ecology Department of Ecological Science Faculty of Earth and Life Sciences VU University Amsterdam Amsterdam The Netherlands; ^2^ Naturalis Biodiversity Center Leiden The Netherlands; ^3^ Department of Zoology School of Biology Aristotle University of Thessaloniki Thessaloniki Macedonia Greece; ^4^ Section of Conservation Biology Department of Environmental Sciences University of Basel Basel Switzerland

**Keywords:** accessory gland protein, allohormone, gastropod, mollusc, morphometry, sexual selection, Stylommatophora

## Abstract

Postcopulatory adaptations that increase reproductive success compared to rivals, like the transfer of accessory gland products that promote paternity, are common when sperm competition occurs among males. In land snails, the dart shooting behavior and its adaptive significance, in promoting individual fitness through enhanced paternity of the successful dart shooter, have been considered such an adaptation. The fitness result gained is mediated by the transfer of mucus components on the love dart capable of altering the physiology of the receiver's reproductive tract. In this context, dart shooting and mucus transfer could be considered as processes targeted by sexual selection. While the effect of dart mucus is beneficial for the dart user, so far it has remained unknown whether its transport is greater when snails experience a higher level of sperm competition. Here, we report results of a study on inter‐ and intraspecific variations of dart and mucus gland morphometry, considered to be traits reflecting the ability of snails to adjust the production and transfer of mucus under varying sperm competition scenarios. We investigated four populations with different densities from four dart‐bearing species, *Arianta arbustorum*,* Cepaea nemoralis*,* Cornu aspersum,* and *Helix lucorum*. The results indicate that different adaptations of these traits occur among the studied species that all seem to achieve the same goal of transferring more mucus when sperm competition is higher. For example, the presence of longer and more branched mucous glands or an increase in dart surface most likely reflect increased mucus production and enhanced ability of mucus transport, respectively. Interestingly, the species for which the use of the dart is reported to be facultative, *A. arbustorum*, did not show any variation among the examined traits. To conclude, sexual selection in the form of sperm competition intensity seems to be an important selective force for these simultaneously hermaphroditic dart‐bearing snails, driving differences in sexual traits.

## 1 INTRODUCTION

When sperm of different males co‐occur in the female reproductive system, the chance of fertilizing all eggs decreases for each male (Parker, 1998). Thus, the generated sperm competition can cause strong selection on postcopulatory adaptations to increase a male reproductive success compared to rivals (Birkhead & Møller, 1998; Simmons, 2001), for example larger testes (e.g., Buzatto, Thyer, Roberts, & Simmons, 2017; Parker, 2016), fast‐swimming sperm, long‐sperm viability (Snook, 2005), and transfer of manipulative accessory gland products (Gillott, 2003). The latter, also termed allohormones (Koene & Ter Maat, 2001), are proteins and peptides that can alter female physiology by causing effects such as the induction of egg‐laying or the delay of female remating (e.g., Chapman & Davies, 2004). In species where the gland products are not transferred together with sperm, they can be injected hypodermically into the partner in different ways (Lange, Reinhardt, Michiels, & Anthes, 2013; Zizzari, Smolders, & Koene, 2014). Examples in separate‐sexed species are the premaxillary teeth of salamanders (Houck, Bell, Reagan‐Wallin, & Feldhoff, 1998) and the stinging organ of scorpions (Tallarovic, Melville, & Brownell, 2000). In simultaneous hermaphrodites, organisms that possess both female and male reproductive organs, examples of such devices are the copulatory setae used by earthworms *Lumbricus terrestris* (Koene, Pförtner, & Michiels, 2005) and the penile stylet of *Siphopteron* sea slugs (Lange, Werminghausen, & Anthes, 2014).

One prominent example of the transfer of accessory gland products in simultaneous hermaphrodites is the love dart of land snails (Tompa, 1984). The love dart is a calcareous stylet that carries such products on its surface, produced by accessory mucous glands. By comparing species, it has been shown that love darts have a species‐specific shape, and darts with an enlarged surface area (e.g., the presence of blades and perpendicular blades on these blades) are associated with more elaborate accessory mucous glands in terms of number and size (Koene & Schulenburg, 2005). During courtship, snails try to stab the dart through the mating partner's body wall, and when they succeed the dart mucus enters the hemolymph of the partner (reviewed by Lodi & Koene, 2016b). The dart mucus, once transferred, causes direct changes in the female reproductive system to the advantage of the dart user (Kimura, Chiba, & Koene, 2014; Kimura, Shibuya, & Chiba, 2013; Koene & Chase, 1998a). This is well‐described for one model species, the brown garden snail *Cornu aspersum* (Koene & Chase, 1998a)*,* where muscular contractions of the spermatophore‐receiving organ, called diverticulum, are induced in vitro. As a result, spermatophore uptake may be aided. Contractions of the copulatory canal are also initiated, causing the closure of the duct entrance leading to the sperm‐digesting organ, called bursa copulatrix (Koene & Chase, 1998a); the latter is an organ that many hermaphrodites use to get rid of excess sperm received from mating partners (e.g., Lodi, Meijer, & Koene, 2017). Via these contractions, more sperm escape digestion and the dart user can roughly double its paternity (Chase & Blanchard, 2006). In other snail species, the dart mucus is also known to cause other effects, like delaying remating of the partner in the bradybaenid *Euhadra quaesita* (Kimura et al., 2013) and a temporary contraction that reduces the length of the diverticulum, probably to favor sperm storage, in the helicid *Eobania vermiculata* (Lodi & Koene, 2016a, 2017).

Most land snail species mate promiscuously and multiple times per reproductive season (Baur, 1998), and within species sperm competition can impose a strong selective force (e.g., Garefalaki, Triantafyllidis, Abatzopoulos, & Staikou, 2010). However, whether differences in level of sperm competition, which is determined by the number of competing ejaculates for egg fertilization (i.e., sperm competition intensity; Parker, Ball, Stockley, & Gage, 1996) and the probability that a male will face sperm competition when mating (i.e., sperm competition risk; Parker, Ball, Stockley, & Gage, 1997), affect reproductive traits of the male function in land snails is not well explored (but see Minoretti & Baur, 2006). In separate‐sexed organisms, there are many cases in which this happens, and that this can result in fixed differences or plastic responses. For example, when there are more competitors, males have an increased testis size (Dziminski, Roberts, Beveridge, & Simmons, 2010; Gage, 1995), highly aggressive behavior (Evans & Magurran, 1999), and increased transfer of accessory gland products (Bretman, Fricke, Hetherington, Stone, & Chapman, 2010). For hermaphrodites, we do know that density can affect paternity success (e.g., Kupfernagel, Rustenholz, & Baur, 2010; Nakadera, Mariën, Van Straalen, & Koene, 2017), and this is often used as a proxy for the potential level of sperm competition. Hypothetically, when snails experience a high level of sperm competition, behavioral adaptations could make dart shooting more accurate in order to successfully transfer manipulative products via the love dart. However, the outcome of this behavior would largely depend on the position of the partner's body with respect to the dart shooter; in helicids the dart successfully hits the partner only 55% of times (Koene & Chase, 1998b). Alternatively, transfer of accessory gland products could be increased by morphological adaptations of the love dart and the associated mucous glands. To test whether morphological adaptations would increase the transfer of dart mucus when sperm competition is strong, in our study we used four dart‐bearing species: *Cornu aspersum*,* Helix lucorum*,* Cepaea nemoralis*, and *Arianta arbustorum* to compare dart surface availability (i.e., dart length, dart blades length, and perimeter of the dart cross‐section) and mucous gland size (i.e., length and number of branches) among four populations with different predicted levels of sperm competition, measured as population density.

## 2 MATERIALS AND METHODS

Adult snails from four populations per species were collected at the beginning of the reproductive period to ensure that the darts would be fully formed and ready to be used. *Cornu aspersum* was collected in July–August in Greece at the localities of Hania (*N *=* *43), Rethymno (*N *=* *34), Kerkyra (*N *=* *36), and Preveza (*N *=* *35). *Helix lucorum* was collected in late April beginning of May in Greece at Axios (*N *=* *31), Gefyra (*N *=* *35), Kokkinopilos (*N *=* *30), and Edessa (*N *=* *32). *Cepaea nemoralis* was collected in the Netherlands in May at the localities of Leiden (*N *=* *24), the Groene Kathedraal in Almere (*N *=* *30), Amsterdamse Bos (*N *=* *21), and Robbenoordbos (*N *=* *40). *Arianta arbustorum* was collected in May in Switzerland at the localities of Moléson (*N *=* *26), Gurnigel (*N *=* *25), Gantrisch (*N *=* *26), and Flumserberg (*N *=* *26). Coordinates for all the collection sites can be found in Table 1. Population density based on adult number was measured with the quadrat method (Staikou, 1998), except for *C. nemoralis*. Since this latter species is distributed on trees (Jaremovic & Rollo, 1979), one person counted snails in a 4 × 10 m area for 30 min (e.g., Schilthuizen, Looijestijn, Chua, Aguirre Gutiérrez, & Castillo Cajas, 2015). For the other three species, we randomly sampled 0.25 m^2^ quadrats to determine the number of adult snails per square meter. Elliott's method was used to determine the number of necessary quadrats to obtain a sampling error less than 20% (Staikou, 1998). After collection, snails were kept at −80°C until dissection. However, after dissection not all individuals had love darts (for sample sizes of each examined trait see Table S1).

**Table 1:** An overview of the origin and simple size of the four species, including their population density and shell volume. Shell volumes that are followed by different letters (a, b, c) are statistically different, within the species, based on post hoc testing (see Results for details)

Species	Country	Population	Coordinates	*N*	Density (indiv./m²)	Shell volume (cmᶟ)
*Arianta arbustorum*	Switzerland	Flumserberg (FL)	47°05′11.9″N 9°16′07.2″E	26	1.0	1.24 ± 0.14 a
		Gurnigel (GU)	46°45′45.3″N 7°27′21.8″E	25	1.2	1.47 ± 0.18 b
		Gantrisch (GA)	46°42′11.7″N 7°26′39.8″E	26	4.4	1.23 ± 0.13 a
		Moléson (MO)	46°33′11.0″N 7°01′44.3″E	26	8.5	1.38 ± 0.17 b
*Cepaea nemoralis*	The Netherlands	Amsterdamse Bos (AMB)	52°19′25.9″N 4°50′52.4″E	21	0.1	2.46 ± 0.34 a
		Groene Kathedraal (GK)	52°19′17.4″N 5°19′00.8″E	30	1.1	2.48 ± 0.28 a
		Leiden (LE)	52°09′29.2″N 4°28′00.8″E	24	2.3	2.26 ± 0.29 a
		Robbenoordbos (RB)	52°54′05.9″N 5°02′59.8″E	40	2.7	2.38 ± 0.36 a
*Cornu aspersum*	Greece	Preveza (PRE)	38°96′87.4″N 20°74′70.5″E	35	6.0	6.10 ± 1.16 a
		Kerkyra (KE)	39°42′63.4″N 19°94′70.2″E	36	8.1	5.54 ± 0.78 b
		Hania (HA)	35°33′56.2″N 24°27′96.5″E	43	10.2	7.98 ± 0.98 c
		Rethymno (RE)	35°34′67.5″N 24°72′82.4″E	34	27.2	6.52 ± 0.84 a
*Helix lucorum*	Greece	Axios (AX)	40°74′73.6″N 22°65′89.3″E	31	6.0	11.09 ± 1.61 a
		Gefyra (GE)	40°73′34.4″N 22°69′57.6″E	35	6.7	9.12 ± 2.13 b
		Kokkinopilos (KO)	40°09′61.1″N 22°25′36.5″E	30	7.7	13.74 ± 1.76 c
		Edessa (ED)	40°79′46.3″N 22°05′73.0″E	32	7.9	9.25 ± 1.38 b

**Figure 1:**
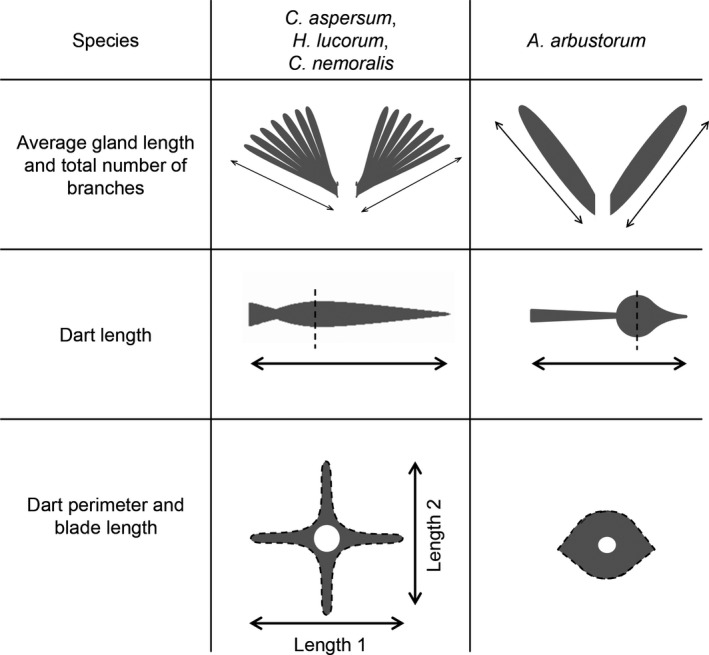
Visualization of the measurements on mucous glands and love darts performed in this study. The species *Arianta arbustorum* is shown in a separate column due to the different shapes of the glands and love dart. Note that in the third row, the perpendicular dashed line represents where the dart was cut to obtain the cross‐sections. In the last row, dart perimeter is indicated by the dashed line

For each snail, the following measurements were conducted. Snail size was estimated by measuring height, length, and width of the shell with a digital calliper after which shell volume was calculated with the formula of Locher and Baur (2000) for both *A. arbustorum* and *C. nemoralis* (Jordaens, De Wolf, Vandecasteele, Blust, & Backeljau, 2006), and the formula of Rogers and Chase (2001) for the other two species. Potential mucus production was estimated by dissecting the mucous glands out and measuring their average length (i.e., snails always have a pair of glands, so the longest branch of each gland was measured and the average calculated), and the total number of branches since these glands are digitiform, except for *A. arbustorum* that has only one branch per gland (Baminger, Locher, & Baur, 2000) (Figure 1). Dart surface availability to carry mucus was estimated by measuring the length of the dart (only when the love dart was complete, meaning that it was fully formed and the tip had not broken off) as well as the perimeter of its cross‐section and the lengths of dart blades of the cross‐section (Figure 1).

**Figure 2:**
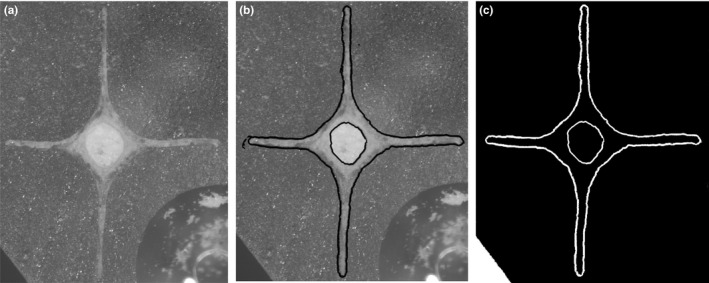
Pictures of a cross‐section of *Cornu aspersum*'s love dart before and after editing. (a) The raw version of the picture taken with the camera connected to a stereo microscope. (b) The image after automatic outlining of the perimeter made with Photoshop. (c) The change in background made with ImageJ, to black, in order to obtain a high contrast with the perimeter

For this purpose, the dart was recovered after the dart sac (i.e., muscular sac containing the dart) was dissected out and dissolved in 2N NaOH over one day (Reyes‐Tur & Koene, 2007). Afterward, the dart was cleaned of tissue remnants and photographed under a stereo microscope connected to a camera. Dart length was measured by ImageJ software. To measure the perimeter of the dart cross‐section, the dart was embedded in transparent resin (^®^Epoxycoating, Polyestershoppen B.V) and cut at the point of its greatest width (where the blades are longest); approximately one‐third of the dart length for the species *Cepaea nemoralis*,* Cornu aspersum,* and *Helix lucorum* (see Figure 1). The dart of *Arianta arbustorum*, which has a completely different shape, was cut at the widest part of the dart tip (see Figure 1). The cuts were performe with a table saw consisting of a circular 0.5 mm wide saw, activated by an electric motor. Then, a picture of the cross‐section was taken with a camera connected to a stereo microscope, and the outline of the dart perimeter was highlighted with the Quick Selection tool in Photoshop. Measurements were made with the ROI manager tool in ImageJ, after the background was darkened to make the contrast with the perimeter more evident (Figure 2). In addition, the lengths of the shorter dart blade (length 1) and the longer blade (length 2) were measured, except for *A. arbustorum* as the dart of this species has no blades (Figure 1). For each species, comparisons of all measured traits were made among populations.

For each trait within each species, we checked whether the data were normally distributed and had homogeneous variance. If this was not the case, a log transformation was applied to reach normality. In order to take body size into account, we used shell volume as a fixed factor in all population analyses. Hence, each species was analyzed using a linear regression model with the fixed factors Population density (indiv./m^2^) and Shell volume (cm^3^) and their interaction and each of the measured traits as response variable.

## 3 RESULTS

### 3.1 Cornu aspersum

Population density (indiv./m²) was 6.0 for the population in Preveza (PRE), 8.1 for Kerkyra (KE), 10.2 for Hania (HA) and 27.2 for Rethymno (RE). Shell volume differed among populations (ANOVA: *F*_3, 144_ **=* *50.289, *p *<* *.0001, on log‐transformed data; Table 1). Post hoc comparisons revealed that snails from population HA were significantly bigger than snails from the other three populations, and snails from population KE were the smallest ones, with PRE and RE not differing from each other but different from both HA and KE (all comparisons based on post hoc Tukey: *p *<* *.05; Table 1).

**Figure 3:**
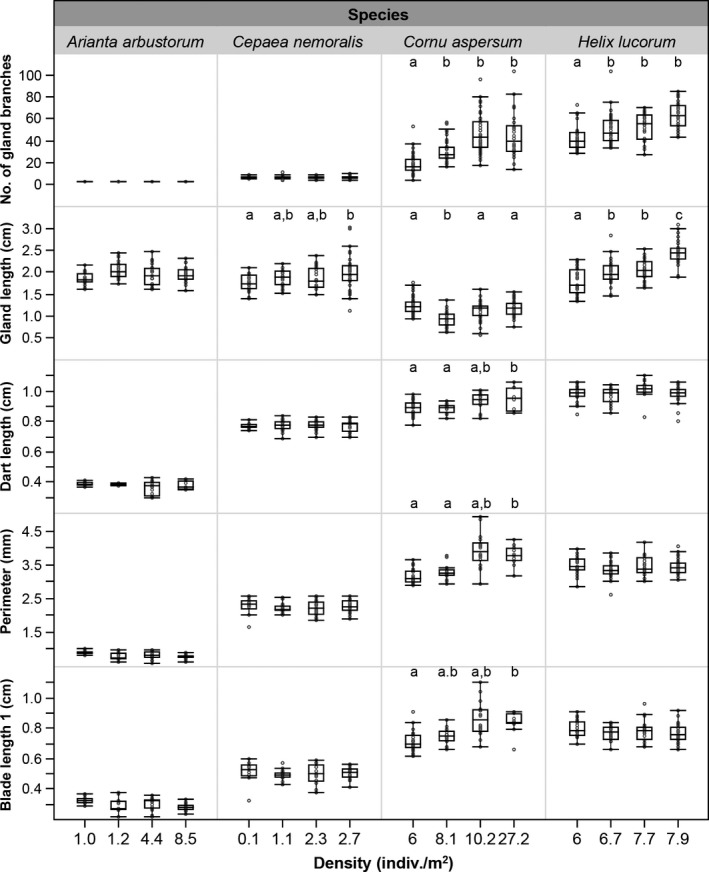
Relationship of population density for the four different species shown with the measured traits of the love dart glands (number of gland branches and gland length) and the dart itself (dart length, perimeter of the cross‐sectioned dart and blade length 1; see methods for details). The boxplots show the mean, quartiles and interquartile range, and the data points are indicated with gray circles. When an overall effect of density was found for a parameter, the statistical differences between the densities are indicated with different letters (a, b, c)

For the gland measurements, the linear regression model revealed that gland length differed with density (*F*_3, 140_ **=* *5.818, *p *=* *.0009), with population KE having shorter glands than the other three (Post hoc Tukey: *p *<* *.05; see Figure 3). Neither shell volume nor its interaction with density had a significant effect. The analysis of the number of gland branches revealed a significant increase with density (log‐transformed*: F*_3, 140_ **=* *31.948, *p *<* *.0001) with individuals from population PRE having a lower number of branches compared to the other populations (Post hoc Tukey: *p *<* *.05; see Figure 3). Again, shell volume and the interaction term had no significant effect.

The dart measurement analysis revealed that dart length increases significantly with density (*F*_3, 45_ **=* *4.844, *p *=* *.005), with the post hoc testing showing that the highest density population (RE) differed significantly from the lowest two (PRE and KE), while HA differed from neither (Post hoc Tukey: *p < *.05; Figure 3). The patterns were the same for the dart perimeter (*F*_3, 59_ **=* *9.366, *p *<* *.0001; post hoc Tukey: *p *<* *.05; Figure 3). Finally, blade length also showed an increase with increasing density (blade length 1: *F*_3, 59_ **=* *5.662, *p *=* *.0018; blade length 2: *F*_3, 59_ **=* *7.182, *p *=* *.0003), with the highest and lowest density populations differing significantly from each other (PRE vs. RE; post hoc Tukey: *p *<* *.05; Figure 3 only shows the values for the short blades, the patterns for the long blades is the same). Shell volume and the interaction term did not have a significant effect in any of the dart measurement models.

### 3.2 Helix lucorum

Population density (indiv./m²) was 6.0 for the population in Axios (AX), 6.7 for Gefyra (GE), 7.7 for Kokkinopilos (KO) and 7.9 for Edessa (ED). Shell volume differed among the four populations (ANOVA: *F*_3, 124_ **=* *43.974, *p *<* *.0001, log‐transformed; Table 1). Individuals from the population KO were found to have the largest volume, significantly differing from the other three populations (Post hoc Tukey: *p *<* *.05). Furthermore, population AX snails were significantly smaller than KO snails but significantly bigger than GE and ED snails (Post hoc Tukey: *p < *.05; Table 1). The volume of the snails from the latter two populations did not differ from each other.

The linear regression model analyzing gland length revealed a significant increase in gland length with density (*F*_3, 120_ **=* *21.475, *p *<* *.0001; Figure 3). Post hoc testing showed that individuals from the population AX had the shortest glands compared to all other populations, whereas individuals from the population ED had the longest glands, with populations GE and KO not differing from each other but differing from both AX and ED (all comparisons: Tukey *p *<* *.05; Figure 3). While shell volume had no significant effect on gland length, the interaction term was significant (Density*Shell volume: *F*_3, 120_ **=* *3.208, *p *=* *.026; Figure 3). The number of gland branches also increased with density (*F*_3, 120_ **=* *13.838, *p *<* *.001, log‐transformed; Figure 3), where individuals from the AX population had significantly fewer branches than the other populations (Post hoc Tukey: *p *<* *.05; Figure 3). Shell volume and the interaction term had no significant effect in this model.

Dart length was found to be marginally different among populations (*F*_3, 75_ **=* *2.458, *p *=* *.069; Figure 3), while shell volume and the interaction term had no significant effect, but post hoc testing revealed no significant difference between the populations (Post hoc Tukey: *p *<* *.05 for all comparisons). Finally, neither the dart perimeter nor the blade measurements seemed to be influenced by density, shell volume or their interaction (Figure 3).

### 3.3 Cepaea nemoralis

Population density (indiv./m²) was 0.1 for the population in Amsterdamse Bos (AMB), 1.1 for Groene Kathedraal (GK), 2.3 for Leiden (LE) and 2.7 for Robbenoordbos (RO). There was no significant difference in body size among the four populations (ANOVA: F_3, 111_ **=* *2.366, *p *=* *.075; Table 1). The only difference found in the measured parameters was a difference in gland length. Gland length increased with population density (*F*_3, 106_ **=* *3.713, *p *=* *.014; Figure 3), with individuals from the RO population having significantly longer glands than those from AMB, while GK and LE differed from neither (Post hoc Tukey: *p *<* *.05; Figure 3). The model did not reveal any significant effect of shell volume and the interaction term.

### 3.4 Arianta arbustorum

Population density (indiv./m²) was 1.0 for the population in Flumserberg (FL), 1.2 for Gurnigel (GU), 4.4 for Gantrisch (GA) and 8.5 for Moléson (MO). Shell volume differed significantly among the four populations (ANOVA: *F*_3, 99_ **=* *14.878, *p *<* *.001, log‐transformed; Table 1). Individuals from the populations MO and GU were significantly bigger than those from GA and FL (Post hoc Tukey: *p *<* *.05). The linear regression models revealed no differences in the gland length, nor in any of the dart measurements (Figure 3). Note that no analysis was performed on the number of gland branches, as snails of this species always have two branches per individual.

## 4 DISCUSSION

We found significant among‐population variation in love dart and accessory mucous gland traits in four simultaneously hermaphroditic land snail species. For three of the four species, gland and dart traits increased with density, with the latter reflecting the potential level of sperm competition (Kupfernagel et al., 2010; Nakadera et al., 2017). These increases are all in line with the idea that they would result in an increase of the transfer of mucous products injected into the mating partner via the dart (although cryptic female choice and sexual antagonism cannot be excluded to play a role here). First, more mucus can be produced by more branched mucous glands and more can be transported by a dart that has more surface available due to longer blades (*C. aspersum*). Second, longer and more branched glands can presumably produce more mucus, which in *H. lucorum* seems to go without clear differences in the dart surface availability (for which the measured dart traits served as a proxy). Third, *C. nemoralis* may produce more mucus in longer glands while its number of gland branches and dart surface availability remain unaltered. So, the general pattern that seems to emerge is that gland morphology (gland length and/or gland number) can change under influence of density. In terms of dart morphology, a clear effect of density on traits that enlarge surface area was only found for *C. aspersum*. Interestingly, the finding that the two lower density populations of *C. aspersum* (PRE and KE), respectively, differ in gland branches and gland number may be indicative of a trade‐off between investing on one or the other, but obviously this needs further investigation.

Overall, the observed trait changes can be advantageous for the dart user as they seem to all have in common that they would result in the transfer of more mucous products into the partner, which would be especially advantageous when sperm competition is strong (i.e., at high density). However, several caveats need to be noted here. Firstly, at the moment there is no empirical evidence indicating that the transfer of more mucus would increase fertilization chances, although the demonstrated in vitro effect of dart mucus is dose‐dependent (Koene & Chase, 1998a). Secondly, the paternity benefit of successful mucus transfer via dart shooting has so far only been demonstrated for *C. aspersum* (Chase & Blanchard, 2006). Hence, it does seem beneficial when more mucus is transported by the love dart, but future research is needed to confirm that this is indeed the case for the other investigated species. Thirdly, it is unknown whether longer glands produce more mucus (containing the love dart allohormone and other bioactive substances; e.g., Stewart, Wang, Koene, Storey, & Cummins, 2016), although there is evidence from research on glands of other species. For example, this is the case for *Drosophila melanogaster*, where males with larger accessory gland size produce more sex peptide, which consequently increases male reproductive success (Wigby et al., 2009).

Among the four dart‐bearing species studied here, the dart and gland traits of *A. arbustorum* did not differ among the populations. Interestingly, *A. arbustorum* has been reported to use its dart facultatively in about 30% of matings (Baminger et al., 2000). As a result, sexual selection pressure may either be weaker in this species or it might target other traits than the ones studied here (see Beese, Beier, & Baur, 2006). Incidentally, this is also in agreement with the recent finding that the effect of the dart mucus of *A. arbustorum* is not as strong as that of other helicids (Lodi & Koene, 2016a) and a previous study indicating that it does not induce the same end result, that is snails hit by a dart did not store more sperm (Bojat & Haase, 2002).

Whether the found differences are the result of fixed, genetic differences or whether these snails can adjust these traits plastically depending on their (developmental) circumstances and population density, remains to be tested. Nevertheless, the differences we find here are in line with predictions based on sexual selection pressures However, as they are relevant in this context, we cannot ignore the potential influence of other (nonsexual) selection pressures and the environment (e.g., Clark, DeBano, & Moore, 1997; Kwiatkowski & Sullivan, 2002; Tomkins, Hazel, Penrose, Radwan, & LeBas, 2011), and most importantly the influence of environmental differences on the strength and form of sexual selection itself (Cornwallis & Uller, 2010). For example, environmental factors may affect mating frequency and favor different adaptations of dart and gland traits among populations. However, despite the fact that all the species were collected from different environments and climate types, a pattern that positively correlates with density still emerges, suggesting that the potential level of sperm competition may be a stronger selective force than the selective forces imposed by the environment and climate. For example, individuals from all populations of *C. aspersum* came from a dry Mediterranean climate where snails estivate all summer (i.e., change occurring during hot periods where snails form a calcareous epiphragm to cover the shell opening after burying themselves into the ground). The individuals from populations RE and HA were collected in natural shrubs and olive trees in Crete, where copulation is restricted to when it occasionally rains during autumn, but mating occurs with several different partners (A. Staikou and coworkers, unpublished data). On the other hand, individuals from populations PRE and KE were sampled in natural shrubs/gardens in the Epirus region (north‐western part of Greece) where snails are exposed to more evenly and continuously distributed rains during autumn and mating frequency is lower than the other two populations (A. Staikou and coworkers, unpublished data). *H. lucorum* snails of the population ED (sampled near waterfalls) and KO (sampled on Olympus mountain) both come from a wet Mediterranean climate (Staikou, Lazaridou‐Dimitriadou, & Farmakis, 1988), while individuals from population GE (from village gardens) and AX (from a natural/trees and shrubs habitat) come from a dry Mediterranean climate. Snails from Axios are the only ones to exhibit estivation for three months compared to the other populations. *A. arbustorum* populations came from a more moist climate, one from a subalpine forest (GU) and the other three from subalpine grassland (Baur & Baur, 1992). Finally, *C. nemoralis* populations were all collected in natural/trees and shrubs habitats in the Netherlands.

Environmental factors such as humidity, temperature, calcareous substrate (Goodfriend, 1986), and plant coverage are also known to affect snail shell size (Baur, 1988). There is generally a positive correlation between body size and male sexual traits (e.g., sperm length in butterflies; Gage, 1994), although these male traits can also coevolve with the morphology of the female reproductive system (e.g., land snails; Beese et al., 2006). In our study, we found very little positive allometry (i.e., correlation of traits with body size), which seems surprising. However, how allometry is influenced by sexual selection is not well known (Bonduriansky & Day, 2003), and this matter becomes even more complex for simultaneous hermaphroditic organisms because body size is a trait shared by the two sex functions (Anthes, 2010). Irrespectively, in our analyses we included body size (as shell volume) to account for possible allometric effects.

In sum, in the investigated simultaneously hermaphroditic land snail species differences in population density, which can be indicative for the level of sperm competition, affected the morphology of reproductive traits that have previously been shown to be important for increasing fertilization chances. This is in line with what is known from research on separate‐sexed species (e.g., Bretman et al., 2010; Dziminski et al., 2010; Evans & Magurran, 1999; Gage, 1995). The variation in morphological traits detected in this study can be advantageous to the dart user as these adaptations can increase a dart shooter's reproductive success when sperm competition is high. Nevertheless, besides these morphological traits, other types of adaptations will also be relevant to investigate. Physiologically, for example, dart mucus composition could be altered to make the paternity enhancing effect more effective (e.g., new manipulative proteins or modification of the existing ones; Eberhard, 1996; Stewart et al., 2016). Consequently, it will now be extremely interesting to test whether mucus quality changes according to the level of sperm competition and whether it affects fertilization success. In addition, antagonistic coevolution is known to occur between the morphology of the spermatophore‐receiving diverticulum and the complexity of the dart in land snails (Koene & Schulenburg, 2005). The diverticulum is longer in species where love darts have an enlarged surface by means of blades and perpendicular blades. Thus, it should also be explored whether the female reproductive system of the high density populations counter‐adapted to the increased efficiency of the dart. All these future studies should ideally be done within a comparative framework in order to uncover different patterns across species, as was done in the current study.

## AUTHOR CONTRIBUTIONS

ML and JMK coordinated the project. JMK obtained the funding for the project. ML, RJ, and AS collected specimens and performed the measurements in the four populations in their respective countries. The preparation of the darts for the analysis was performed jointly in Amsterdam with all co‐authors. ML and JMK did the statistical analysis, which was subsequently checked by RJ and AS. The manuscript was prepared by ML, after which the other co‐authors provided feedback and additional information.

## CONFLICT OF INTEREST

None declared.
