# Supplementary material for: High level of sperm competition may increase transfer of accessory gland products carried by the love dart of land snails
Source: Ecol Evol. 2017 Nov 17;7(24):11148–56. doi: 10.1002/ece3.3385 (PMC5743536; doi:10.1002/ece3.3385)
Supplement: Supplementary file 1 [file ECE3-7-11148-s001.docx]

**Table S1** An overview of the samples size of each trait examined in the four land snail species.

| Species | Population | Shell volume  (cmᶟ) | Average gland length (cm) | Total number of gland branches | Dart  length (mm) | Dart perimeter (mm) | Dart blade length 1 & 2 (mm) |
| --- | --- | --- | --- | --- | --- | --- | --- |
| *A. arbustorum* | FL | 26 | 21 | 21 | 9 | 9 | n.a. |
|  | GU | 25 | 20 | 20 | 4 | 9 | n.a. |
|  | GA | 26 | 21 | 21 | 15 | 13 | n.a. |
|  | MO | 26 | 20 | 20 | 7 | 12 | n.a. |
| *C. nemoralis* | AMB | 21 | 21 | 21 | 9 | 10 | 10 |
|  | GK | 30 | 30 | 30 | 21 | 19 | 19 |
|  | LE | 24 | 24 | 24 | 18 | 17 | 17 |
|  | RB | 40 | 40 | 40 | 18 | 17 | 17 |
| *C. aspersum* | PRE | 35 | 35 | 35 | 23 | 24 | 24 |
|  | KE | 36 | 36 | 36 | 15 | 16 | 16 |
|  | HA | 43 | 43 | 43 | 16 | 17 | 17 |
|  | RE | 34 | 34 | 34 | 8 | 11 | 11 |
| *H. lucorum* | AX | 31 | 31 | 31 | 19 | 21 | 21 |
|  | GE | 35 | 35 | 35 | 21 | 24 | 24 |
|  | KO | 30 | 30 | 30 | 24 | 26 | 26 |
|  | ED | 32 | 32 | 32 | 19 | 22 | 22 |
